# Olfactory impairment in first-episode schizophrenia: a case-control study, and sex dimorphism in the relationship between olfactory impairment and psychotic symptoms

**DOI:** 10.1186/s12888-018-1786-8

**Published:** 2018-06-18

**Authors:** Xiacan Chen, Jiajun Xu, Bin Li, Wanjun Guo, Jun Zhang, Junmei Hu

**Affiliations:** 1 West China School of Basic Medical Sciences & Forensic Medicine, Sichuan University, Sichuan, China; 2 Mental Health Center, West China Hospital, Sichuan University, Sichuan, China

**Keywords:** Schizophrenia, Olfaction, Sex, Negative symptom, Positive symptom

## Abstract

**Background:**

A body of studies has focused on the olfactory impairment among people with schizophrenia. The effect of sex on this relationship has attracted the attention of researchers. These issues have not been studied much in Chinese schizophrenia patients.

**Methods:**

We conducted a case-control study of 110 first-episode antipsychotic medicine naïve schizophrenia patients aged 18–35 years and 110 controls, matched by age and sex. Odour threshold, discrimination and identification were assessed by the “Sniffin’ Sticks” test. Psychotic symptoms were assessed by the Positive and Negative Syndrome Scale (PANSS).

**Results:**

The odour threshold, discrimination and identification scores of patients with schizophrenia were significantly lower than those of the healthy control group. The difference in identification score had statistical significance between male and female patients with schizophrenia (*t* = − 2.45, *P* < 0.05). Controlling for confounding factor, in male schizophrenia participants, the negative subscale score was significantly and inversely correlated with the discrimination (γ = − 0.37, *p* < 0.008), identification (γ = − 0.45, *p* < 0.008) and TDI (γ = − 0.50, *p* < 0.008) scores; the general psychopathology subscale score was inversely and significantly correlated with the identification (γ = − 0.47, *p* < 0.008) and TDI (γ = − 0.41, *p* < 0.008) scores. For female schizophrenia patients, positive and general psychopathology subscale scores had a significant inverse correlation with the identification score (positive: γ = − 0.47, p < 0.008; general psychopathology: γ = − 0.42, *p* < 0.008).

**Conclusions:**

Controlling for confounder, negative symptoms were related to impaired odour discrimination and identification in male schizophrenia patients, while positive symptoms were correlated with impaired odour identification in female schizophrenia patients. This sex dimorphism could provide useful information for future studies aiming to finding biomarkers of schizophrenia.

## Background

Schizophrenia, mainly defined by its psychotic symptoms, lacks physiological examination to diagnosis [1]. Many studies were conducted with the aim of finding biomarkers of schizophrenia and olfactory function has been one of the directions. Olfaction, an age- and sex-related function, is one of the most direct neural pathways between external environment and the central nervous system. There is overlap of some neuroanatomical structure with the impaired brain region in those with schizophrenia, such as the medial temporal lobe, amygdala and hippocampus [1–3]. Furthermore, the olfactory function examination was more convenient and reliable than a review of the patient symptom state which is subjective and always reported by patients themselves. Lastly, olfactory impairment was found in schizophrenia patients, and has been especially correlated with negative symptoms [4, 5]. Consequently, an understanding olfaction would be useful for the clinical practice of schizophrenia.

Olfaction is a multiple sensory process, and the deficit of it can be divided into peripheral and central abnormalities. Odour detection threshold (sensitivity) is more peripheral, while identification and discrimination are more central [6–8]. Olfactory dysfunction is well documented in schizophrenia patients and odour identification impairment has been suggested as a vulnerability marker of deficit syndrome in some studies [5, 9], while a meta-analysis failed to find evidence for considering olfaction identification as vulnerability marker of schizophrenia [10]; however, the absence of accounting for sex effect in the meta-analysis could profoundly influence the results, for sex difference in olfaction have been documented not only in healthy individuals, but also in schizophrenia patients [11, 12]. Many studies were conducted with small sample sizes and these multiple olfactory functions (e.g., threshold, identification and discrimination) were barely examined in same subjects, as *Moberg* noted [13]; in addition, odour threshold and discrimination were reported much less among patients with schizophrenia when comparing with odour identification [4, 7].

Sexual dimorphism is common in neuroscience and schizophrenia studies. The differences of manifestations, social function and brain structure have been addressed between male and female among schizophrenia [14–17]. Sex difference was also presented in olfactory performance among patients with schizophrenia, in that some researchers have reported that male schizophrenia patients show a strong relation between negative symptoms and odour identification, while female patients have failed to present this relation [12, 18]. However, the relation between psychotic symptoms and other odour domains (e.g., threshold and discrimination) has been rarely studied.

Meanwhile, olfaction was reported as an age-related function among general adults, but many studies did not include age as a confounding factor when detecting olfactory impairment among patients with schizophrenia [11, 19]. Even though many studies about olfactory impairment among patients with schizophrenia have been conducted, few studies aimed at reporting the relation between olfaction and schizophrenia in Chinese patients. Addressing these limitations, we want to conduct a case-control study with the aim of detecting three olfactory performances in Chinese schizophrenia patients compared with healthy control subjects, and elucidating the sexual dimorphism in the relationship between psychotic symptom and olfactory function among schizophrenia patients, with the consideration of age effect, to provide useful information for further olfactory study in schizophrenia and clinical practice.

## Methods

### Procedure and participates

The aim of this study was to detect differences in responses to three olfactory performances in Chinese schizophrenia patients compared with healthy control subjects and to examine the sexual dimorphism in the relationship between psychotic symptoms and olfactory function among schizophrenia patients, with the consideration of age effect.

For this study, we recruited 110 patients aged 18–35 years old who were diagnosed with schizophrenia (66 males, 44 females). The patients were diagnosed according to DSM-V; patients were excluded for neurological disorder, organic brain injury, any other mental illness, any major olfactory disturbance histories or general poor somatic health. These participants were all in the first-episode stage of schizophrenia and naive to any antipsychotic medication prior to the olfactory testing. Young adults (18–35 years old) were selected as participants for two reasons: one was that in previous studies of the “Sniffin’ Sticks” test (SST) in a general population, a difference was found in olfactory performance among different adult age groups (e.g., young adult, middle-aged adult and older adult) [11, 19]; the other reason was that first-episode schizophrenia is most often seex in the young adult (18–35 years old) was the main age group.

Healthy controls, 110 community-based subjects aged 18–35 years, were recruited by advertising and were free of any psychotic disorders according to DSM-V. The exclusion criteria were in accordance with the schizophrenia group; additionally, individuals with a family history of psychosis were excluded. Age (±3 years) and sex were matched for each control. The demographic details are shown in Table 1. All healthy participants and guardians of patients provided written informed consent.

**Table 1:** Demographic characteristics and olfactory performance scores

	Schizophrenia	Healthy	*t*
	*n* = 110 (male: 66;female:44)	n = 110 (male:66;female:44)	
Age, mean (SD) in years	22.86 (4.61)	23.46 (4.18)	5265 (Mann-Whitney)
M	22.85 (4.69)	24.47 (4.80)	1737*
F	22.89 (4.56)	22.00 (2.40)	967
Sex, N (p)			
M	66	66	0 (χ^2^)
F	44	44	
Smoking	21 (19.1)	8 (7.3)	6.7*(χ^2^)
M	19 (28.79)	8 (12.12)	5.63*(χ^2^)
F	2 (4.54)	0	2.05(χ^2^)
SST, mean (SD) in score			
Threshold	5.98 (2.77)	8.75 (3.18)	6.89^**^
M	5.92 (3.08)	8.92 (3.28)	5.42^**^
F	6.07 (2.25)	8.50 (3.05)	4.26^**^
Discrimination	8.73 (3.55)	12.44 (1.79)	9.80^**^
M	8.62 (3.82)	12.55 (1.76)	7.58^**^
F	8.89 (3.13)	12.27 (1.84)	6.20^**^
Identification	10.60 (2.73)	13.02 (1.72)	7.12^**^
M	10.09 (3.00)	13.14 (1.76)	6.99^**^
F	11.36 (2.09)	12.84 (1.66)	3.67^**^
TDI	25.31 (6.65)	34.21 (4.98)	11.23^**^
M	24.64 (7.24)	34.61 (5.09)	9.15^**^
F	26.32 (5.58)	33.61 (4.81)	6.57^**^

*SST* the Sniffin’ sticks test, *M* Male, *F* Female, *TDI* the total score of threshold, discrimination and identification

*: *P* < 0.05; **: *P* < 0.01

### Psychotic symptom assessment

Psychotic symptoms were assessed by two psychiatrists using the Positive and Negative Syndrome Scale (PANSS) and the interrater reliability was 0.81. This scale included positive, negative and general psychopathy subscales [20].

### Olfactory testing

The Sniffin’ Sticks Test, with a high repeatability, was used to evaluate olfaction [21, 22]. It is composed of three tests—odour threshold, odour discrimination and odour identification. The odour threshold task consists of 16 types of n-butanol solution with different concentrations, which start from 4%. Triplet of felt-tipped pens is a group with two containing blank solvent and a third containing the odorant of n-butanol solution, and the triplets are presented to the subject in randomized order. The subject is asked to determine the odorant from the other two blank solvent.

The odour discrimination task comprises 16 groups as well. A group, consisting of a triplet of pens, includes two with the same odorant and a third with a different odorant. A triplet of pens is randomly presented to the subject. The subject has to identify which one smelled different.

During the odour identification test, 16 odours were presented to participants through the felt-tipped pen dispensers and each odour must be identified by the subject from a list of four descriptors.

In the three sub-tests, the interval between the presentation of each pen and each group was approximately 3 s and 20–30 s, respectively. As the score of each sub-test ranges from 0 to 16 and the total score, which was the TDI score comprising threshold, discrimination and identification, ranges from 0 to 48.

### Statistical analyses

Continuous data were described by mean or median and compared between the schizophrenia and control groups by *t* test, or non-parametric test if the data did not meet normal distribution. The Kolmogorov–Smirnov test was used to assess the normality. Categorical data were compared by chi-square test between the two groups. Partial correlation was used to control confounding factors and show the relationship between clinical scales (PANSS) and the SST scores. An alpha level of below 0.05 was defined as statistical significance. The significant *P* value was corrected by Bonferroni Correction for multiple testing. All data analyses were performed with the statistical package SPSS (version 16.0).

## Results

**Fig. 1:**
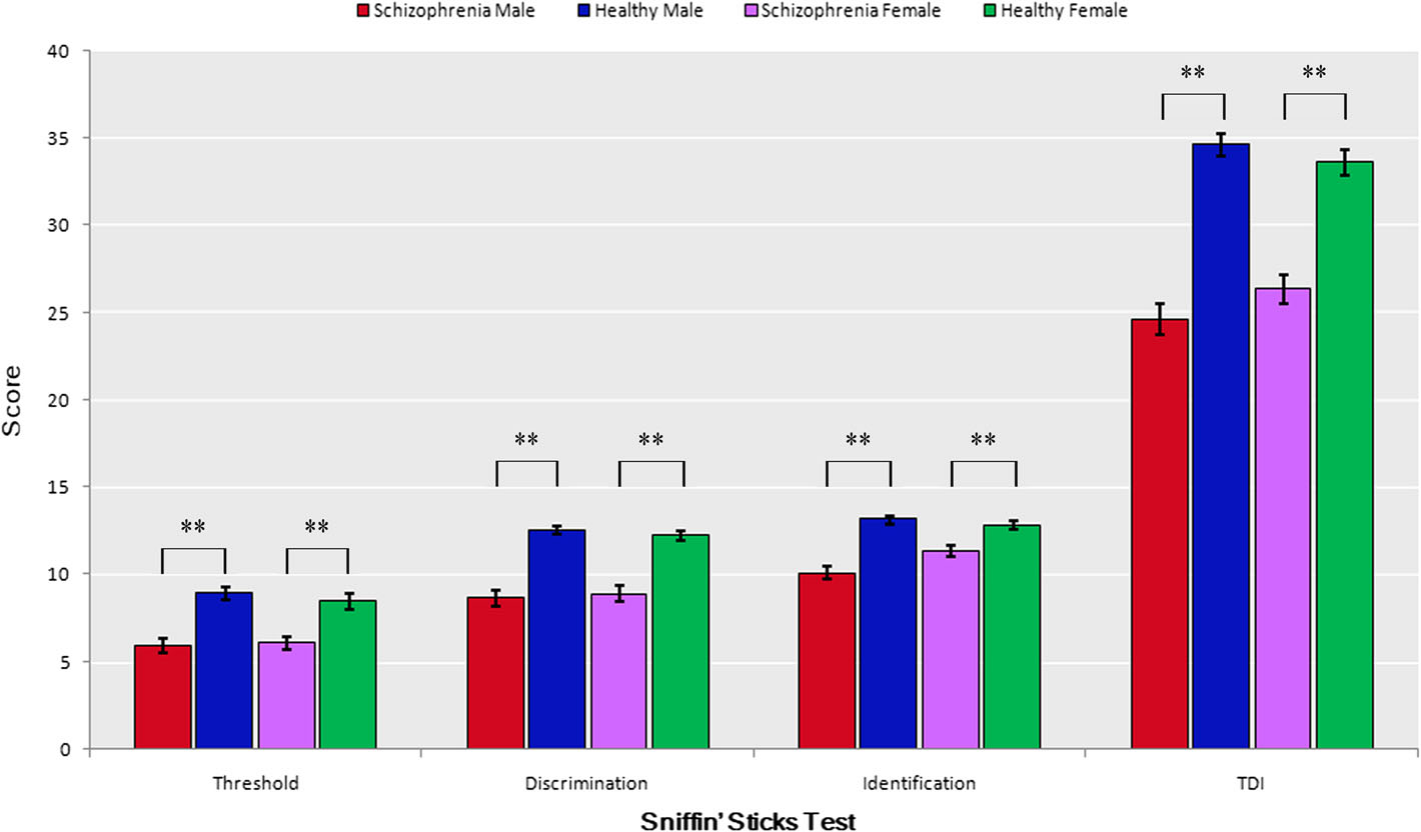
Mean “Sniffin’ Sticks” test scores with standard error bars by diagnosis and sex. Abbreviations TDI, the total score of threshold, discrimination and identification

Demographic factors, smoking status and the SST scores (including olfactory threshold, discrimination, identification and TDI score) are displayed in Table 1. The average age was 22.86 (SD = 4.61) and 23.46 (SD = 4.18) years for the schizophrenia and healthy group, respectively. There was no significant difference in the age and sex between the schizophrenia and healthy group. The schizophrenia participants showed lower mean scores of SST test than the control group among both males and females (Table 1 and Fig. 1). Among the schizophrenia group, the difference in smoking status shows statistical significance between male and female (χ^2^ = 10.01, *P* < 0.01).

The olfactory performances and psychotic symptoms for male and female schizophrenia patients are illustrated in Table 2. The positive, negative and general psychopathology subscale scores, and the five factor symptom scores did not show a statistical difference; the difference of the identification score had statistical significance (*t* = − 2.45, *P* < 0.05), while the threshold, discrimination and TDI scores did not.

**Table 2:** Comparison of PANSS scores and the SST scores between male and female schizophrenia

	Male	Female	*t*
	*n* = 66	*n* = 44	
SST score, mean (SD)			
Threshold	5.92 (3.08)	6.07 (2.25)	−.27
Discrimination	8.62 (3.82)	8.89 (3.13)	−.38
Identification	10.09 (3.00)	11.36 (2.09)	−2.45^*^
TDI	24.64 (7.24)	26.32 (5.58)	−1.30
PANSS score, mean (SD)			
Sub-scale score			
Positive	16.26 (4.97)	15.82 (4.60)	.47
Negative	16.80 (6.22)	15.89 (5.23)	.81
General Psychopathy	31.76 (8.40)	30.52 (7.49)	.79

*PANSS* the positive and negative syndrome scale, *SST* the Sniffin’ sticks test, *TDI* the total score of threshold, discrimination and identification

^*^: *P* < 0.05; ^**^: *P* < 0.01

**Fig. 2:**
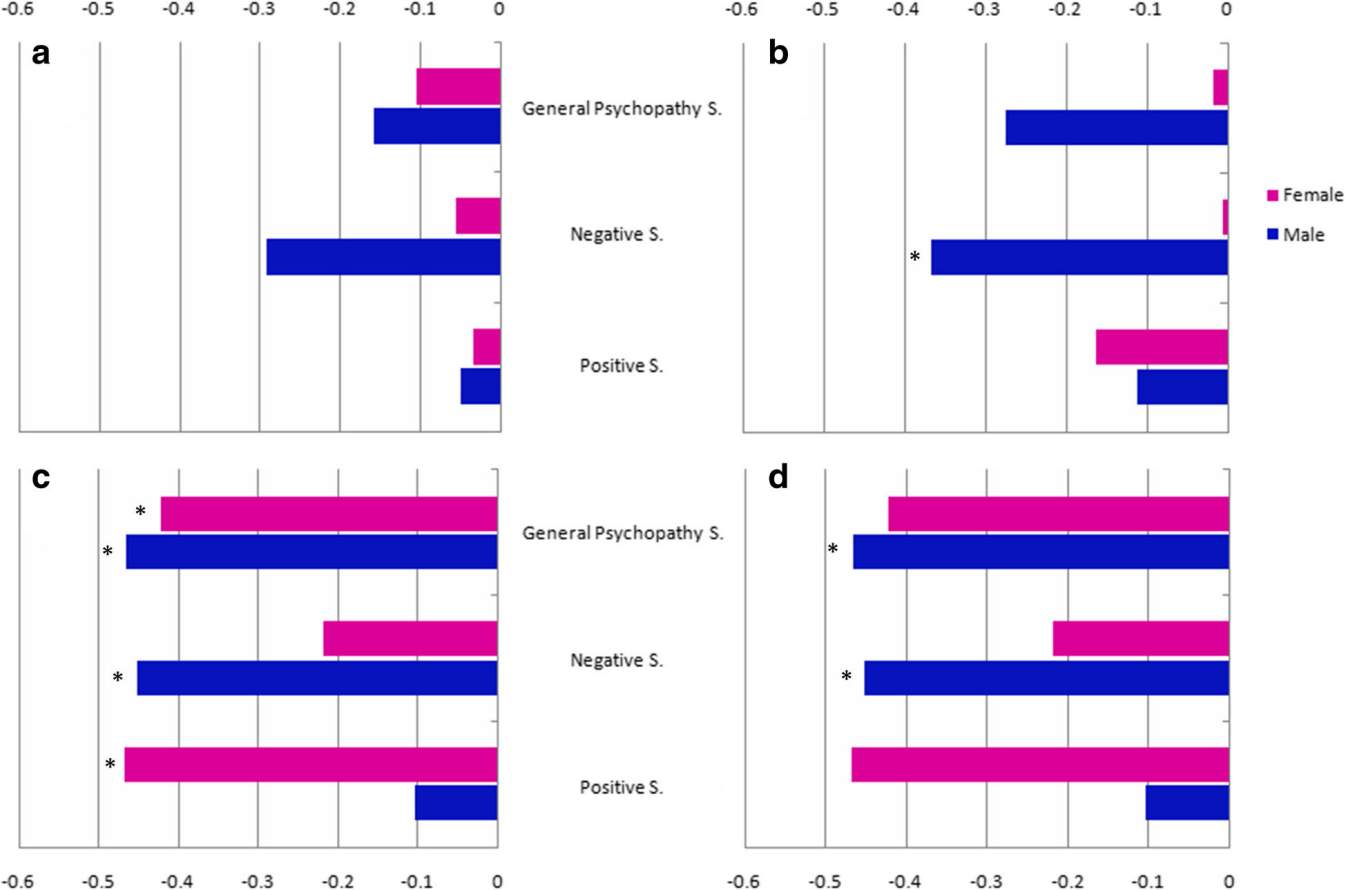
Partial correlations between olfaction and psychotic symptoms in schizophrenia group. **a**: Partial correlations between odour threshold score and PANSS scores; **b**: Partial correlations between odour discrimination score and PANSS scores; **c**: Partial correlations between odour identification score and PANSS scores; **d**: Partial correlations between TDI and PANSS score. Adjusted for the smoking status. Abbreviations: General psychopathy S., general psychopathy subscale; Negative S., negative subscale; Positive S., positive subscale. PANSS, the Positive and Negative Syndrome Scale; TDI, the total score of threshold, discrimination and identification.*: Adjusted *P* < 0.008

The threshold (KS Z = 1.69, *p* < 0.05), discrimination (KS Z = 1.85, *p* < 0.05), identification (KS Z = 1.75, *p* < 0.05) and TDI (KS Z = 1.14, *p* < 0.05) scores did not meet the normal distribution, nor did the positive symptom score (KS Z = 1.38, *p* = 0.046). The negative symptom score met the normal distribution (KS Z = 0.94, *p* = 0.35). Correlation between psychotic symptoms and olfactory performance in the schizophrenia group is illustrated in Fig. 2. Controlling for the confounding factor, in male schizophrenia participants, the negative subscale score showed a significant inverse correlation with discrimination (γ = − 0.37, *p* < 0.008), identification (γ = − 0.45, *p* < 0.008) and TDI (γ = − 0.50, p < 0.008) scores; the general psychopathology subscale score was inversely and significantly correlated with identification (γ = − 0.47, *p* < 0.008), and the TDI (γ = − 0.41, *p* < 0.008) scores.

In contrast, among female schizophrenia patients, positive and general psychopathology subscale scores appeared to have a significant inverse correlation with the identification score (positive: γ = − 0.47, *p* < 0.008; general psychopathology: γ = − 0.42, *p* < 0.008).

## Discussion

Considering the effect of age, we conducted this case-control study to explore the olfactory function of first-episode schizophrenia patients aged 18–35 in China and the sex effect on the relationship between olfaction and schizophrenia with the consideration of the smoking confounder. In this study, olfactory impairment was found among Chinese schizophrenia patients. In terms of psychotic symptoms, negative symptoms were correlated with odour discrimination and identification in male schizophrenia patients, whereas positive symptoms were correlated with odour identification in female schizophrenia patients. General psychopathy symptom was correlated with odour identification both in male and female schizophrenia patients.

In this study, matched by sex and age, schizophrenia patients showed poorer performance across all the measures of olfaction, compared with the healthy group. Although many researchers have reported olfactory impairment in schizophrenia since 20 years ago [23], few have reported about olfactory performance among those with schizophrenia. Recently, a study conducted in China reported an odour identification deficit in patients with schizophrenia [24]; unfortunately, this study only detected the odour identification performance instead of the whole olfactory function of Chinese patients with schizophrenia.

After adjusting for the smoking status, odour identification was correlated with negative and positive symptoms among male and female schizophrenia patients, respectively. The extant literature had noted a robust correlation between odour identification and negative symptoms in schizophrenia [25, 26] and many studies further showed sex effect on olfactory function both in schizophrenia [27, 28] and in general population [11, 22]. Further studies considering sex effect indicated a high correlation between negative symptoms and odour identification only in male schizophrenia patients [29]. Our study is strongly consistent with this finding that poorer odour identification was only related to negative symptoms in male schizophrenia patients. In addition, a different finding that olfactory identification was correlated with positive symptoms among female schizophrenia patients still remains in this study. This was scarcely reported before. Only a recently study found fewer serious positive symptoms were associated with better odour detection threshold for citralva [30].We think whether considering the effect of sex or not might account for the different reporting. Moreover, because the negative symptom of schizophrenia have been reported to be correlated with olfactory impairment by researchers starting two decades ago [13], many following studies have focused on exploring negative symptoms and might neglect positive symptoms when detecting olfactory function in patients with schizophrenia [12].

Meanwhile, sex dimorphism exists in the symptomatology, antipsychotic response [28], social functioning [14], illness characteristics [15] and cerebral structure [16] of schizophrenia. It might be helpful to consider these factors, as well as hormone effect [28], when future studies try to elucidate the sex effect on olfaction. Meanwhile, odour identification has been described as a central process [31] and the limbic structure (e.g., amygdala and the orbitofrontal cortex) was the location of olfactory association cortex. The abnormality of limbic structure was not only connected with olfactory dysfunction [31] but also connected with negative symptomatology [32]. Furthermore, the orbitofrontal cortex to amygdala ratio presented sex dimorphism [33], and hyperfrontality and hypofrontality was correlated with positive and negative symptoms, respectively [34, 35]. Collectively, these might provide another possible explanation for the sex difference presented regarding the relationship between olfactory impairment and symptomatology. Moreover, the sex related difference of olfactory bulb [36] might also be a possible way to elucidate this result. In addition, Chinese patients were participants in this study, for whom the sex effect on olfaction was hardly reported. It might be possible that there is heterogeneity of olfactory impairment in Chinese schizophrenia patients.

Odour discrimination, studied much less than olfactory identification in previous research [8], was related to negative symptoms among male patients in this study. Odour discrimination being more central processing when compared with odour threshold, and its deficit is related to the hippocampus and amygdala, which are also the impaired brain region among schizophrenia [31]. Therefore, the relation between odour discrimination and negative symptoms in male schizophrenia patients should be detailed further. Expectedly, general psychopathy, including cognition, was correlated with odour identification. This was in consistent with previous studies that have found that olfactory identification was strongly related with cognition and it was considered to be a useful supplementary screening tool of Alzheimer’s disease [37].

The limitation of this study was the lack of neuroimaging or electrophysiological studies to further explain why different sex shows different relationships between psychotic symptoms and olfactory impairment. This is the direction of our further studies.

## Conclusion

Sex influenced the relationship between psychotic symptoms and olfactory impairment in first-episode schizophrenia patients aged 18–35 years. Negative and positive symptoms were associated with odour identification deficit in male and female schizophrenia, respectively. The odour discrimination deficit was correlated with negative symptom among male schizophrenia patients. The effect of sex on olfactory impairment among patients with schizophrenia should be studied more profoundly.

## Abbreviations

PANSS: Positive and Negative Syndrome Scale
SST: The Sniffin’ Sticks Test
TDI: The total score of threshold, discrimination and identification
